# Different Infection Profiles and Antimicrobial Resistance Patterns Between Burn ICU and Common Wards

**DOI:** 10.3389/fcimb.2021.681731

**Published:** 2021-06-30

**Authors:** Yali Gong, Yuan Peng, Xiaoqiang Luo, Cheng Zhang, Yunlong Shi, Yixin Zhang, Jun Deng, Yizhi Peng, Gaoxing Luo, Haisheng Li

**Affiliations:** ^1^ Institute of Burn Research, Southwest Hospital, Third Military Medical University (Army Medical University), Chongqing, China; ^2^ State Key Laboratory of Trauma, Burns and Combined Injury, Chongqing, China; ^3^ Department of Plastic and Reconstructive Surgery, Shanghai Ninth People’s Hospital, Shanghai JiaoTong University School of Medicine, Shanghai, China

**Keywords:** burn infection, antimicrobial resistance, multi-drug resistance, carbapenem-resistant Gram-negative bacteria, methicillin-resistant *Staphylococcus aureus*, fungi

## Abstract

Infection is the leading cause of complications and deaths after burns. However, the difference in infection patterns between the burn intensive care unit (BICU) and burn common wards (BCW) have not been clearly investigated. The present study aimed to compare the infection profile, antimicrobial resistance, and their changing patterns in burn patients in BICU and BCW. Clinical samples were analyzed between January 1, 2011, and December 31, 2019, in the Institute of Burn Research in Southwest China. The patient information, pathogen distribution, sources, and antimicrobial resistance were retrospectively collected. A total of 3457 and 4219 strains were detected in BICU and BCW, respectively. Wound secretions accounted for 86.6% and 44.9% in BCW and BICU, respectively. Compared with samples in BCW, samples in BICU had more fungi (11.8% vs. 8.1%), more Gram-negative bacteria (60.0% vs. 50.8%), and less Gram-positive bacteria (28.2% vs. 41.1%). *Acinetobacter baumannii* were the most common pathogen in BICU, compared with *Staphylococcus aureus* in BCW. *S. aureus* was the most frequent pathogen in wound secretions and tissues from both BICU and BCW. However, *A. baumannii* were the first in blood, sputum, and catheter samples from BICU. Overall, the multidrug-resistance (MDR) rate was higher in BICU than in BCW. However, the gap between BICU and BCW gradually shortened from 2011 to 2019. The prevalence of MDR *A. baumannii* and *Klebsiella pneumonia* significantly increased, especially in BCW. Furthermore, Carbapenem resistance among *K. pneumoniae* significantly increased in BICU (4.5% in 2011 vs. 40% in 2019) and BCW (0 in 2011 vs. 40% in 2019). However, the percentage of MDR *P. aeruginosa* sharply dropped from 85.7% to 24.5% in BICU. The incidence of MRSA was significantly higher in BICU than in BCW (94.2% vs. 71.0%) and stayed at a high level in BICU (89.5% to 96.3%). *C. tropicalis* and *C. albicans* were the two most frequent fungi. No resistance to Amphotericin B was detected. Our study shows that the infection profile is different between BICU and BCW, and multidrug resistance is more serious in BICU than BCW. Therefore, different infection-control strategies should be emphasized in different burn populations.

## Introduction

Infection is the most common complication and the leading cause of death in burn patients (Jeschke et al., 2020). About 38.27% of burn deaths were caused by systemic infection in China. Burn patients are predisposed to infection because of the loss of skin barrier protection and the acquired immunosuppression. The diagnosis of infection depends on physical examination, infection biomarker detection, and microbiology culture. Antibiotic use and wound care are two important aspects of infection control (Jeschke et al., 2020). However, the first use of antibiotics is usually performed without microbiological results, mostly based on the epidemiology of microbiology. Therefore, it is crucial to investigate the pathogen distribution, antimicrobial resistance, and their changing patterns to direct antimicrobial Prescription and reduce antimicrobial misuse.

Numerous studies show that burn infection is positively related with burn severity, such as burn area, burn depth, inhalation injury, and burn severity scores. Therefore, different infection control and treatment methods should be utilized with severe and nonsevere burn patients. The Institute of Burn Research in the Southwest Hospital of the Third Military Medical University is one of the longest operating burn centers in China and largest burn centers in the world; it has 125 inpatient beds (including 18 ICU beds) and specializes in burn care and treatment. In our center, severe burn patients, who have large burn areas (children: >30%, adults: >50%), suffered >10% total body surface area (TBSA) of full-thickness burns, need tracheotomy or mechanical ventilation, or are combined with inhalation injury or complicated with other injuries, are enrolled in the burn intensive care unit (BICU), which is on a separate floor from common wards. In a 1-year preliminary study, we previously found that the profile and antibiotic resistance of microorganisms in the BICU were obviously different from those in burn common wards (BCW) (Yali et al., 2014). However, the sample size was relatively low, and many new strategies of wound care, such as MEEK grafts, negative pressure wound therapy, and artificial skin, have been widely applied in recent years (Li et al., 2017). Furthermore, pathogen distribution and antimicrobial resistance might have changed a lot in the past nine years. As a result, it is urgent to investigate the changes in microbiology and the infection profile of burn patients and confirm the differences between the BICU and BCW.

## Materials and Methods

### Study Design and Ethical Considerations

This was a retrospective study performed between January 1, 2011, and December 31, 2019, in the Institute of Burn Research, the Southwest Hospital of the Third (Army) Military Medical University. This study was approved by the ethical committee of the Southwest Hospital (No. KY201991).

### Data Collection

Data were collected from the burn microbiology laboratory in our institute and from medical records. Four microbiology technicians and three burn care specialists reviewed all the data. The following data were extracted: demographic data (gender, age), clinical features (burn etiology, burn area), sample sources, microbe type, and antimicrobial resistance. To avoid the influence of antibiotics on the pattern of drug resistance, only the results of the first positive isolates were included in this study as previously described (Polemis et al., 2020). A repeated result of the same pathogen from the same sample source of the same patient was excluded. However, the same pathogen from a different sample source of the same patient was included.

### Microbe Species Identification and Antimicrobial Susceptibility Test

All growth microbes were classified into Gram-negative bacteria, Gram-positive bacteria, and fungi and identified based on standard microbiological procedures. Blood, chocolate, MaiKangKai, and Sarpaul Petri dishes were used for the culture and inoculation of microbial samples. Different types of samples were inoculated in the corresponding Petri dishes and incubated in the corresponding incubators for 18 to 24 h as required and then taken out for the observation of colony morphology. After isolating and purifying the bacterial strains, identification and drug sensitivity experiments were carried out with VITEK-2 compact system analysis. Detailed steps are described in the reference (Yali et al., 2014).

The drug sensitivity test was conducted according to the Clinical and Laboratory Standards Institute (CLSI) document M100. The minimum inhibitory concentration (MIC) method was employed in this study. A total of 27 antibacterial drugs were used in this study, including ampicillin (10 μg), piperacillin (100 μg), amoxicillin/clavulanate (20/10 μg), cefoperazone/subactam (30/75 μg), ampicillin/sulbactam (10/10 μg), piperacillin/tazobactam (100/10 μg), cefoperazone (75 μg), ceftazidime (30 μg), ceftriaxone (30 μg), cefotaxime (30 μg), cefepime (30 μg), aztreonam (30 μg), imipenem (10 μg), amikacin (30 μg), gentamicin (120 μg for *genus enterococcus*, 10 μg for others), tobramycin (10 μg), ciprofloxacin (5 μg), levofloxacin (5 μg), SMZ-TMP (1.25/23.75 μg), polymyxin B (300 μg), tigecycline (15 μg), oxacillin (1 μg), rifampicin (5 μg), moxifloxacin (5 μg), ofloxacin (5 μg), clindamycin (2 μg), erythromycin (15 μg), linezolid (30 μg), vancomycin (30 μg), teicoplanin (30 μg), chloramphenicol (30 μg), quinuptin/dafoptin (15 μg), tetracycline (30 μg), and minocyline (30 μg). High-level gentamicin was only used in the sensitivity analysis of *genus enterococcus* to predict the synergistic effects of aminoglycoside antibiotics combined with ampicillin, penicillin, or vancomycin as indicated in CLSI M100. Six kinds of antifungal drugs were selected, including voriconazole, amphotericin B, fluconazole, itraconazole, ketoconazole, and 5-fluorocytosine. The break point of bacteria was determined by reference to CLSI M100, and the break point of fungus was determined by reference to CLSI M60. For the methicillin-resistant *S. aureus* (MRSA) detection method, cefoxitin was used to detect MRSA according to CLSI M100 requirements. The MIC value of cefoxitin ≤4 was MSSA, and ≥8 was MRSA. *S. aureus* ATCC 25923, *E. coli* ATCC 25922, and *P. aeruginosa* ATCC 27853 were used as internal quality control. Carbapenem-resistant *Enterobacteriaceae* (CRE) is defined as being resistant to imipenem. Multidrug resistance (MDR) is designated as resistance of a pathogen to > 3 classes of common antimicrobial agents (Falagas and Karageorgopoulos, 2008).

### Statistical Analysis

Data were primarily input and processed using Microsoft Excel 2016 (USA, Microsoft), and descriptive statistics (mean, percentage, standard deviation) were calculated. Data analysis was performed using WHONET5.5 software (World Health Organization), GraphPad Prism 6 (USA, GraphPad Software Inc.), and SPSS 19.0 (USA, IBM analytics). The chi-square test was applied to assess significant associations between two categorical variables. Student’s *t*-test was used to compare two means of quantitative variables. *P* values <.05 were considered statistically significant.

## Results

### Clinical Feature of Burn Patients

A total of 23,717 samples from 1159 burn patients in BICU and 22,672 samples from 14,379 burn patients in BCW were analyzed from January 2011 to December 2019. Furthermore, 3457 pathogens were detected in the 23,717 specimens from BICU (14.6%), and 4219 pathogens were detected in the 22,672 specimens from BCW (18.6%). The clinical feature of burn patients in BICU and BCW are shown in **Table 1**. Patients in BICU had more TBSAs and older ages than those in common wards (TBSA: 58.9 ± 12.3 vs. 13.7 ± 7.3, *P* <.001; age: 43.8 ± 23.2 vs. 28.3 ± 22.8, *P* <.001).

**Table 1:** Clinical feature of burn patients in BICU and BCW.

	BICU	BCW
**Cases (n)**	1159	14,379
**Total Samples (n)**	23,717	22,672
**Positive samples (n)**	3457	4219
**Ages (Mean ± SD)**	43.8 ± 23.2*	28. 3 ± 22.8
**Gender**		
Males (n, %)	735 (63.4)	9284 (64.6)
Females (n, %)	424 (36.6)	5095 (35.4)
**Etiology**		
Flame (n, %)	607 (52.4)	2062 (14.3)
Scald (n, %)	289 (24.9)	5279 (36.7)
Electrical burns (n, %)	95 (8.2)	1624 (11.3)
Others (n, %)	168 (14.5)	5414 (37.7)
**TBSA (mean ± SD)**	58.9 ± 12.3*	13.7 ± 7.3

*P <.001 compared with patients in BCW.

### Pathogen Sources

**Figure 1:**
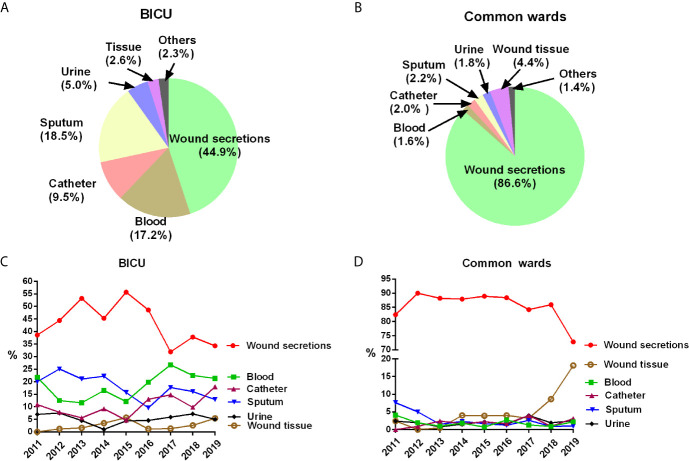
Pathogen sources in BICU and BCW. **(A)** Pathogen sources in BICU. **(B)** Pathogen sources in BCW. **(C)** The annual kinetics of pathogen sources in BICU. **(D)** The annual kinetics of pathogen sources in BCW.

Different diseased regions had great differences in the specimen sources (**Figure 1** and **Table 2**). Although wound secretions accounted for the highest percentage in both BICU and BCW, the values varied greatly (44.9% in BICU and 86.6% in BCW). Accordingly, other sample sources showed different patterns. In BICU, the percentage of sputum, blood, and catheter were secondary to that of wound secretions, comprising 18.5%, 17.2%, and 9.5%, respectively (**Figure 1A**). In BCW, the percentage of other sources were similar, ranging from 1.4% to 4.4% (**Figure 1B**). Furthermore, the kinetics of sample sources were also different in BICU and BCW (**Figures 1C, D**). In BICU, the percentage of blood samples gradually increased from 2011 to 2019 and have ranked in the second position since 2016. In BCW, the percentage of samples collected from wound tissue greatly elevated from about 4% before 2017 to 18.1% in 2019.

**Table 2:** Pathogen distributions among different types of clinical samples.

Pathogens	Wounds (%)		Blood (%)		Sputum (%)		Catheters (%)		Urine (%)	
	BICU(*n* = 1642)	BCW (*n* = 3837)	BICU(*n* = 595)	BCW (*n* = 67)	BICU(*n* = 639)	BCW (*n* = 91)	BICU(*n* = 328)	BCW (*n* = 84)	BICU(*n* = 173)	BCW (*n* = 78)
**Gram+ bacteria**	**36.5**	**42.4**	**27.7**	**47.8**	**16.9**	**23.1**	**23.5**	**57.1**	**8.1**	**9.0**
*S. aureus*	23.0	26.8	13.8	19.4	13.6	15.4	17.1	52.4	1.7	0.0
*S. haemolyticus*	3.5	3.5	1.7	9.0	0.2	2.2	2.7	4.8	1.2	0.0
*E. faecium*	2.7	2.8	3.7	4.5	0.3	2.2	1.8	1.2	4.0	14.1
**Gram- bacteria**	**59.6**	**52.1**	**63.2**	**37.3**	**70.0**	**51.6**	**61.6**	**29.8**	**33.5**	**61.5**
*A. baumannii*	15.6	6.7	24.7	9.0	26.1	13.2	23.8	11.9	6.9	5.1
*P. aeruginosa*	14.3	15.4	12.6	9.0	13.6	14.3	22.3	14.3	13.3	12.8
*K. pneumoniae*	5.8	4.4	5.5	3.0	9.9	8.8	6.4	1.2	9.2	9.0
*E. cloacae*	4.4	5.4	4.4	3.0	1.7	4.4	2.4	0.0	0.6	1.3
*E. coli*	5.8	6.5	1.7	3.0	1.6	0.0	3.0	1.2	7.5	15.4
*S.maltophilia*	2.9	1.5	3.0	0.0	4.5	0.0	2.1	0.0	0.0	2.6
**Fungi**	**6.5**	**7.3**	**8.6**	**11.9**	**15.2**	**22.0**	**13.1**	**13.1**	**64.2**	**26.9**
*C. albicans*	1.6	0.9	1.2	7.5	7.0	12.1	2.1	3.6	9.2	7.7
*C. tropicalis*	1.5	1.0	1.2	0.0	3.3	1.1	2.1	4.8	16.2	6.4
*C. parapsilosis*	1.0	0.9	2.9	3.0	0.8	0.0	4.3	3.6	8.7	5.1
*C. glabrata*	0.4	0.5	1.0	0.0	1.9	4.4	1.5	0.0	10.4	0.0

BICU, Burn intensive care units; BCW, burn common wards. The bold values mean the percentage of Gram+ bacteria, Gram- bacteria and fungi of all the pathogens in different clinical samples.

### Infection Profile

#### Pathogen Types in BICU and BCW

**Figure 2:**
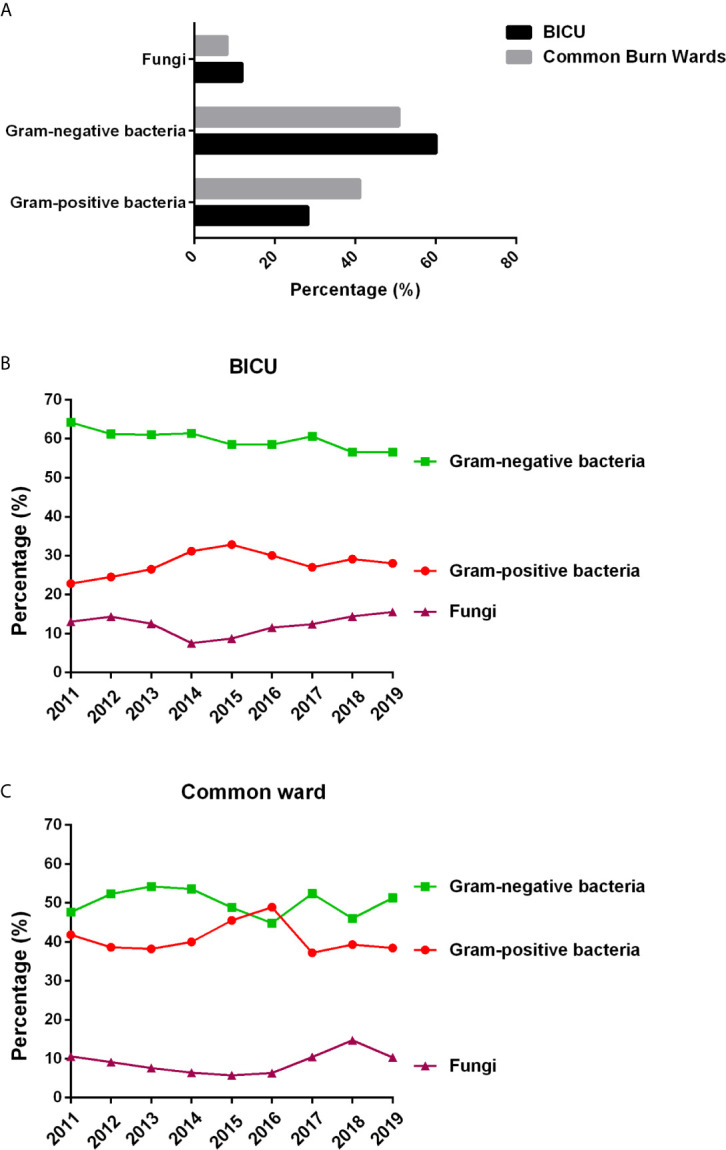
Pathogen types in BICU and BCW. **(A)** The composition of different type of pathogens. **(B)** The changes of pathogen composition in BICU during 2011 to 2019. **(C)** The changes of pathogen composition in BCW during 2011 to 2019.

Gram-negative bacteria were the primary pathogens in burn patients from both BICU and BCW (**Figure 2A**). Of the 3457 pathogens from BICU, 60.0% were Gram-negative bacteria, 28.2% were Gram-positive bacteria, and 11.8% were fungi. Of the 4219 pathogens from BCW, 50.8% were Gram-negative bacteria, 41.1% were Gram-positive bacteria, and 8.1% were fungi. Compared with samples in BCW, samples in BICU had a higher percentage of Gram-negative bacteria and fungi and a lower percentage of Gram-positive bacteria. During 2011 to 2019, the percentage of Gram-negative bacteria ranged from 56.5% to 64.2% in BICU samples and from 44.8% to 54.2% in BCW samples (**Figures 2B, C**).

#### Main Pathogens in BICU and BCW

**Figure 3:**
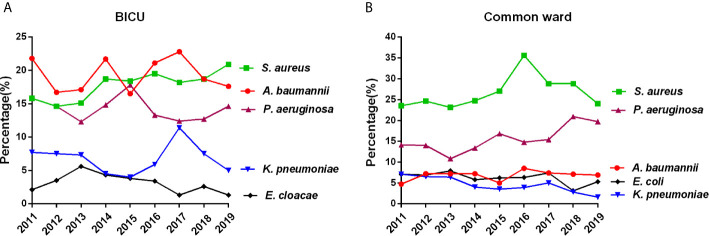
Main pathogens in BICU and BCW. Changes of the five most common pathogens in BICU **(A)** and BCW **(B)**.

As shown in **Figure 3** and **Tables S1**, **S2**, the top 10 pathogens in BICU were *Acinetobacter baumannii, Staphylococcus aureus, Pseudomonas aeruginosa, Klebsiella pneumoniae, Enterobacter cloacae, Candida albicans, Escherichia coli, Enterococcus faecium, Staphylococcus haemolyticus*, and *Candida tropicalis*. The top 10 pathogens in BCW were *Staphylococcus aureus, Pseudomonas aeruginosa, Acinetobacter baumannii, Klebsiella pneumoniae, Escherichia coli, Enterobacter cloacae, Staphylococcus haemolyticus, Proteus mirabilis, Enterococcus faecium*, and *Staphylococcus hominis*. In BICU, the rate of *S. aureus* gradually increased from 15.8% in 2011 to 20.9% in 2019 and became the highest one in 2019 (**Table S1**). In BCW, the percentage of *S. aureus* continued to be highest, and the percentage of *P. aeruginosa* gradually increased from 14.1% in 2011 to 19.7% in 2019 (**Table S2**).

#### Microbiome Composition by Specimen Type in BICU and BCW

Previous studies show that microbiome composition might be different by specimen type. Therefore, we analyzed the pathogen distribution among different types of samples in BICU and BCW (**Table 2**). Gram-positive bacteria were dominant in blood and catheter samples from patients in BCW compared with Gram-negative bacteria in BICU. Furthermore, Gram-negative bacteria were predominant in wound and sputum samples from patients in BCW and BICU. Fungi are the main pathogens in urine samples from patients in BICU, but Gram-negative bacteria are primary in urine samples from patients in BCW. Overall, *S. aureus*, *A. baumannii*, and *P. aeruginosa* were the three main bacteria in wounds, blood, sputum, and catheters in patients from BICU and BCW. In the same sample type, the percentage of *A. baumannii* and *P. aeruginosa* were higher in patients from BICU than in patients from BCW, but the percentage of *S. aureus* was higher in patients from BCW than in patients from BICU. In detail, *S. aureus* was the most frequent pathogen in clinical samples from wounds in both BICU and BCW. Furthermore, *S. aureus* were the first in blood, sputum, and catheters from patients in BCW. Nevertheless, *A. baumannii* was the first in blood, sputum, and catheter samples from patients in BICU. *C. tropicalis* was the most common pathogen in urine samples from patients in BICU compared with *E. coli* in BCW.

### Antimicrobial Resistance

#### The Annual Change of Common MDR Bacteria

**Figure 4:**
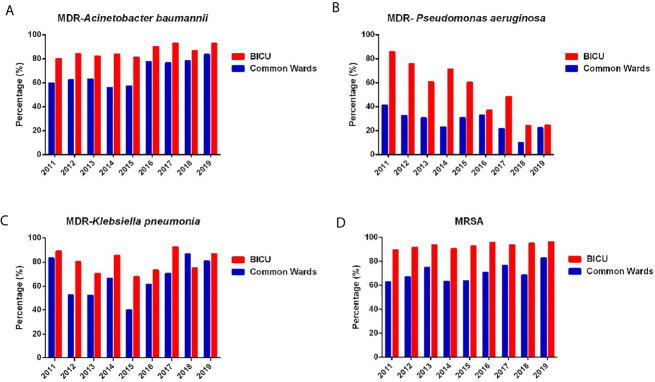
Annual changes of MDR of common bacteria. **(A)** Annual distribution of MDR of *Acinetobacter baumannii* from BICU and BCW. **(B)** Annual distribution of MDR of *Pseudomonas aeruginosa* from BICU and BCW. **(C)** Annual distribution of MDR of *Klebsiella pneumonia* from BICU and BCW. **(D)** Annual distribution of MDR of *MRSA* from BICU and BCW.

The prevalence of MDR bacteria has become a major global public health problem because of high mortality and poor effective antibiotics. The annual changes in MDR *P. aeruginosa*, *A. baumannii*, *K. pneumonia*, and MRSA are shown in **Figure 4**. Overall, the percentage of MDR bacteria in BICU were higher than those in BCW. However, the gap between BICU and BCW has gradually shortened from 2011 to 2019. The prevalence of MDR *A. baumannii* significantly increased in BICU (80.2% in 2011 vs. 92.7% in 2019) and BCW (59.7% in 2011 vs. 89.3% in 2019) (**Figure 4A**). In BICU, the occurrence of MDR *P. aeruginosa* sharply dropped from 85.7% in 2011 to 24.5% in 2019. A decrease in the percentage of MDR *P. aeruginosa* was also found in BW (23.5% in 2011 vs. 6.9% in 2019) (**Figure 4B**). Since 2015, the rate of MDR *K. pneumoniae* also increased in BICU (67.7% in 2015 vs. 86.8% in 2019) and BCW (40.0% in 2015 vs. 80.7% in 2019) (**Figure 4C**). The incidence of MRSA was significantly higher in BICU than in BCW (94.2% vs. 71.0%). The occurrence rate of MRSA stayed at a high level in our cohorts, ranging from 89.5% to 96.3% in BICU and 62.8% to 82.9% in BCW (**Figure 4D**).

#### Antimicrobial Resistance of Gram-Negative Bacteria

**Figure 5:**
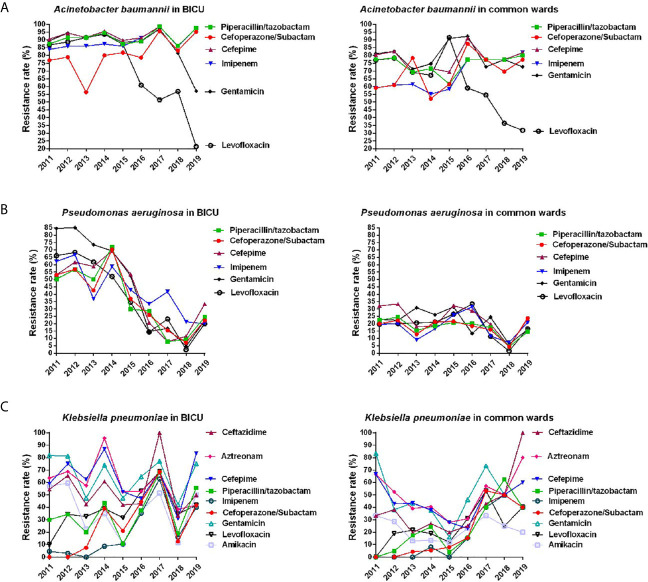
Annual changes of antibacterial resistance of main Gram-negative bacteria. **(A)** Annual distribution of antibacterial resistance of *Acinetobacter baumannii* from burn ICU (left) and common ward (right). **(B)** Annual distribution of antibacterial resistance of *Pseudomonas aeruginosa* from burn ICU (left) and common ward (right). **(C)** Annual distribution of antibacterial resistance of *Klebsiella pneumonia* from burn ICU (left) and common ward (right).

The most common types of Gram-negative bacteria in BICU and BCW were *P. aeruginosa*, *A. baumannii*, *K. pneumoniae*, *E. coli*, and *E. cloacae.* As shown in **Table 3**, all of them were completely sensitive to Tigecycline and Polymyxin B. The occurrence rate of Carbapenem-resistant Gram-negative bacteria were significantly higher in BICU than in BCW. The resistance of *A. baumannii* from both BICU and BCW were higher than that of other Gram-negative bacteria. The annual changes of antimicrobial resistance are shown in **Figure 5** and **Tables S3–S5**.

**Table 3:** Antimicrobial resistance of Gram-negative bacteria in BICU and BCW.

Pathogens	*A. baumannii*		*P. aeruginosa*		*K. pneumoniae*		*E. cloacae*		*E. coli*	
	BICU(*n* = 660)	BCW (*n* = 288)	BICU(*n* = 492)	BCW (*n* = 630)	BICU(*n* = 228)	BCW (*n* = 186)	BICU(*n* = 119)	BCW (*n* = 214)	BICU(*n* = 138)	BCW (*n* = 265)
Ampicillin	ND	ND	ND	ND	98.7	97.4	86.5	87.5	94.3	88.9
Piperacillin	94.0	74.9	55.5	15.0	83.3	53.7	67.0	36.1	84.0	84.7
Amoxicillin/clavulanate	ND	ND	ND	ND	77.9	30.0	91.3	95.7	70.5	34.8
Cefoperazone/Subactam	80.1	61.0	42.7	18.3	29.4	14.7	21.9	4.6	2.9	5.4
Ampicillin/sulbactam	90.5	69.8	ND	ND	76.4	46.9	82.6	77.0	63.2	60.0
Piperacillin/tazobactam	92.7	71.3	34.7	11.5	35.1	12.7	28.9	10.7	7.4	11.6
Cefoperazone	ND	ND	ND	ND	74.0	45.9	55.7	26.2	76.6	65.6
Cefuroxime	ND	ND	ND	ND	78.3	51.1	73.9	64.8	77.3	65.7
Ceftazidime	91.6	72.7	37.6	18.3	51.9	23.9	60.8	26.6	47.8	38.6
Ceftriaxone	95.0	84.1	ND	ND	70.8	70.0	31.8	28.6	79.5	62.7
Cefotaxime	93.4	80.5	98.6	97.1	77.1	50.0	69.1	36.1	78.7	64.6
Cefepime	93.2	72.5	39.4	15.0	58.9	38.2	48.9	19.1	75.0	52.5
Aztreonam	99.5	98.7	38.4	25.9	62.2	38.9	22.7	29.2	50.0	53.6
Imipenem	93.2	58.2	42.3	12.4	22.7	2.3	8.8	0.6	2.5	2.2
Amikacin	87.5	68.9	37.4	7.4	36.4	14.5	25.2	11.7	19.3	19.9
Gentamicin	92.1	73.1	42.5	13.5	64.0	35.4	42.3	23.0	64.8	53.0
Tobramycin	90.7	71.4	49.8	11.1	58.5	36.6	42.8	31.5	63.6	25.4
Ciprofloxacin	93.7	70.7	25.8	8.3	57.3	33.6	25.8	13.0	65.9	59.7
Levofloxacin	89.5	45.5	35.5	7.9	40.9	17.6	14.9	8.0	65.9	55.8
SMZ-TMP	89.0	69.2	99.1	99.0	71.6	50.4	49.4	37.7	78.4	58.2
Polymyxin B	0	0	0	0	ND	ND	ND	ND	ND	ND
Tigecycline	0	0	ND	ND	0	0	0	0	0	0

BICU, Burn intensive care units; BCW, burn common wards; ND, Not done.

As shown in **Figure 5A** and **Table S3**, the rate of *A. baumannii* resistance to common antibiotics in BICU was slightly higher than those in BCW. For BICU, the resistant rate of *A. baumannii* was 83.8%–98.6% to Imipenem and 56.4%–95.7% to Cefoperazone/Subactam. In BCW, the resistant rate of *A. baumannii* to Imipenem greatly increased from 59.1% in 2011 to 81.8% in 2019. The rate of *A. baumannii* resistance to Levofloxacin rapidly decreased in the BICU and BCW since 2015. The changes of antimicrobial resistance of *P. aeruginosa* are shown in **Figure 5B** and **Table S4**. Overall, the resistance rate of *P. aeruginosa* to common antibiotics obviously decreased from 50.2%–84.5% in 2011 to 20%–33.3% in 2019 in BICU and steadily stayed at less than 35% in BCW during 2011 to 2019. Since 2017, the resistance rate of *P. aeruginosa* became similar between BICU and BCW. Furthermore, the spectrum of antimicrobial resistance showed no obvious difference between different antibiotics.

The overall antimicrobial resistance of *K. pneumoniae* was more severe in BICU than that in BCW before 2016 (**Figure 5C** and **Table S5**). However, the resistance rate seemed close after 2016 because it was increased in BCW. In both BICU and BCW, the resistant rate of *K. pneumoniae* to imipenem significantly increased from about 2.5% during 2011–2013 to about 40% during 2017–2019. This phenomenon could also be found in cefoperazone/subactam sensitivity. The resistance rate was second lowest for amikacin and was relatively highest for aztreonam and third-generation cephalosporins, such as ceftazidime and cefepime. The mean resistance rate of *K. pneumoniae* to levofloxacin was 24.52% in BCW and 38.38% in BICU.

#### Antimicrobial Resistance of Gram-Positive Bacteria

As mentioned, *S. aureus*, *S. haemolyticus*, and *E. faecium* are the three main types of Gram-positive bacteria in our cohort. As shown in **Table 4**, no *S. aureus*, *S. haemolyticus*, or *E. faecium* strains were resistant to vancomycin, teicoplanin, linezolid, and tigecycline. On the whole, the resistance rate of the three Gram-positive bacteria were higher in BICU than in BCW to common antibiotics, such as gentamycin, levofloxacin, tetracycline, rifampicin, ciprofloxacin, moxifloxacin, quinuptin/dafoptin, and chloramphenicol. The resistance of *S. haemolyticus* and *E. faecium to* sulfamethoxazole, clindamycin, and erythromycin were significantly higher than those of *S. aureus*. However, the resistance rate of *S. aureus* was higher than *S. haemolyticus* and *E. faecium* to chloramphenicol, quinuptin/dafoptin, and tetracycline.

**Table 4:** Antimicrobial resistance of Gram-positive bacteria in BICU and BCW.

Pathogens	*S. aureus*		*S. haemolyticus*		*E. faecium*	
	BICU (*n* = 606)	BCW (*n* = 1099)	BICU (*n* = 79)	BCW (*n* = 148)	BICU (*n* = =81)	BCW (*n* = 125)
Ampicillin	ND	ND	ND	ND	80.0	63.2
Penicillin	100	98.0	100	100	ND	ND
Oxacillin	94.2	71.0	94.1	98.2	ND	ND
Gentamycin (120 ug)#	ND	ND	ND	ND	55.0	50.0
Gentamycin (10 ug)	94.4	65.3	82.4	79.3	ND	ND
Rifampicin	93.4	57.0	31.7	30.6	89.1	71.1
Ciprofloxacin	94.4	95.4	93.3	87.4	80.0	47.4
Levofloxacin	94.4	59.0	94.1	96.7	75.0	66.7
Moxifloxacin	93.9	57.6	94.1	63.3	80.0	83.3
Ofloxacin	94.2	64.1	93.3	88.3	ND	ND
SMZ-TMP	2.3	9.3	61.7	51.7	ND	ND
Clindamycin	18.1	47.3	53.8	76.9	ND	ND
Erythromycin	49.6	75.1	93.3	96.4	94.4	84.2
Linezolid	0	0	0	0	0	0
Vancomycin	0	0	0	0	0	0
Teicoplanin	0	0	0	0	0	0
Tigecycline	0	0	0	0	0	0
Chloramphenicol	78.7	49.6	30.0	27.9	1.8	7.9
Quinuptin/Dafoptin	82.0	52.7	14.3	7.7	ND	ND
Tetracycline	95.8	67.5	52.9	37.9	61.8	16.7
Minocyline	ND	ND	ND	ND	40.0	44.7

BICU, Burn intensive care units; BCW, burn common wards; ND, Not done. #: High-level gentamicin was only used in sensitivity analysis of genus enterococcus to predict the synergistic effects of aminoglycoside antibiotics combined with ampicillin, penicillin, or vancomycin as indicated in CLSI M100.

**Figure 6:**
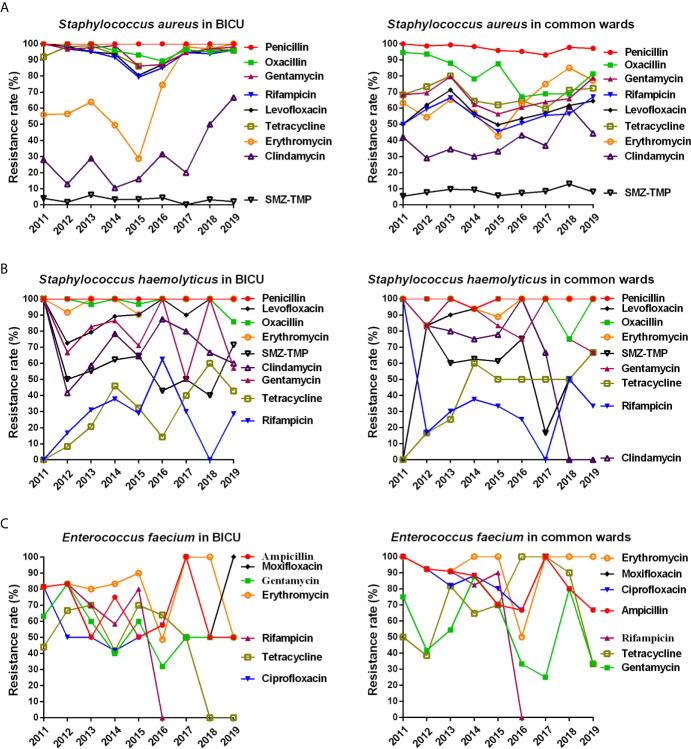
Annual changes of antibacterial resistance of main Gram-positive bacteria. **(A)** Annual distribution of antibacterial resistance of *Staphylococcus aureus* from burn ICU (left) and common ward (right). **(B)** Annual distribution of antibacterial resistance of Staphylococcus haemolyticus from burn ICU (left) and common ward (right). **(C)** Annual distribution of antibacterial resistance of Enterococcus faecium from burn ICU (left) and common ward (right).

The annual distribution of antimicrobial resistance is shown in **Figure 6** and **Tables S6–S8**. As shown in **Figure 6A** and **Table S6**, the resistant rate of *S. aureus* was higher in BICU (79.0%–100%) than in BCW (45.6%–94.7%) to gentamycin, levofloxacin, tetracycline, and rifampicin. A minority of *S. aureus* was tolerant to SMZ-TMP. The rate of *S. aureus* resistance to erythromycin and clindamycin rose rapidly in both BICU and BCW from 2011 to 2019. The resistance of *S. haemolyticus* to tetracycline and rifampicin increased in BICU and BCW during 2011 to 2019 (**Figure 5B** and **Table S7**). However, the clindamycin resistance of *S. haemolyticus* in BCW decreased from 100% in 2011 to 0% in 2018 and 2019. Furthermore, the resistance rate to other antibiotics fluctuated. The resistant rate of *E. faecium* to high-level gentamycin fluctuated around 50% in BICU and BW (**Figure 5C** and **Table S8**). *E. faecium* in both BICU and BCW showed high resistance to moxifloxacin and erythromycin of about 80% to 90%.

### Fungi

A total of 408 isolates in BICU were fungi, accounting for 11.8% of positive isolates, and 341 isolates in BCW were fungi, about 8.1% of positive isolates. The percentage of patients with fungal infection complicated with bacterial infection were 38.7% in BCW and 72.1% in BICU. Furthermore, 12.3% and 53.2% of patients had fungal infection complicated with *S. aureus* infection in BCW and BICU, respectively. Clinical samples from wounds, urine, and sputum were the main sources of fungi, comprising 51.7%, 17.6%, and 15.6%, respectively. *C. albicans*, *C. tropicalis*, *C. parapsilosis*, and *C. glabrata* were the most common type of fungi in our cohort, accounting for 21.4%, 18.3%, 14.8%, and 9.5%, respectively. *C. albicans* were the most frequent fungi in BICU (101/408, 24.8%) and BCW (59/341, 17.3%), followed by *C. tropicalis* in BICU (87/408, 21.3%) and BCW (50/341, 14.7%). As shown in **Table 5**, no resistance to amphotericin B was detected, but the resistance rate of *C. tropicalis* and *C. glabrata* were generally higher than *C. albicans* and *C. parapsilosis*, especially to ketoconazole, fluconazole, and 5-Fluorocytosine. Most *C. albicans* and *C. parapsilosis* were not tolerant to common antifungal drugs. The resistance rate of *C. tropicalis* were relatively lower to ketoconazole and 5-fluorocytosine than voriconazole and fluconazole. Voriconazole were the most sensitive agents for treating *C. glabrata*, secondary only to amphotericin B.

**Table 5:** The resistance of common fungi to antifungal drugs.

Pathogens	*Candida albicans*		*Candida tropicalis*		*Candida parapsilosis*		*Candida glabrata*	
	BICU (*n* = 101)	BCW (*n* = 59)	BICU (*n* = 87)	BCW (*n* = 50)	BICU (*n* = 68)	BCW (*n* = 43)	BICU (*n* = 48)	BCW (*n* = 23)
Voriconazole	1	0	13.7	5.3	1.1	2.4	4.5	0
Amphotericin B	0	0	0	0	0	0	0	0
5-Fluorocytosine	4.8	0	0	0	2.4	0	50.0	47.9
Fluconazole	0	1.7	19.2	10.5	0	0	63.0	48.2
Itracoazole	0	0	10.7	5.3	0	0	54.5	33.3
Ketoconazole	2.7	0	3.2	0	0	0	28.7	16.8

BICU, Burn intensive care units; BCW, burn common wards.

## Discussion

Infection has always been a great challenge of burn treatment, and MDR has become a global health concern in recent years. This study confirms that infection profiles showed different patterns between patients in BICU and BCW. First, sample sources were different. Most samples were collected from wound secretions in BCW (86.6%), nearly double those in BICU (44.9%). Pathogen types also differed by sample sources. Second, pathogen distribution showed different characteristics. Compared with samples in BCW, samples in BICU had a higher percentage of Gram-negative bacteria and fungi and a lower percentage of Gram-positive bacteria. In particular, *A. baumannii*, *S. aureus*, and *P. aeruginosa* were the three most common pathogens in BICU compared with *S. aureus*, *P. aeruginosa*, and *A. baumannii* in BCW. Third, the prevalence of MDR bacteria, such as *S. aureus*, *P. aeruginosa*, *A. baumannii*, and *K. pneumoniae*, are higher in BICU than in BCW. This study also found that Carbapenem-resistant *K. pneumoniae* (CRKP) and *A. baumannii* significantly increased from 2011 to 2019 in both BICU and BCW, but Carbapenem-resistant *P. aeruginosa* obviously decreased from 62.2% to 20% in BICU. Last, fungal susceptibility was lower in BICU than that in BCW although fungal distribution was similar between BICU and BCW. Therefore, different infection control strategies should be emphasized in different burn populations.

This study confirms that microorganisms in BICU and BCW show different patterns. Burn patients in BICU had higher infection rates, stronger resistance, and a higher percentage of Gram-negative bacteria and fungi than patients in BCW. The following reasons contribute to this phenomenon. First, patients in BICU have more predisposing factors to infection than patients in BCW. Compared with patients in BCW, burn patients in BICU often have more burn area, deeper burn depth, present inhalation injury, longer wound healing time, compromised immune function, multiple organ dysfunction, and longer length of stay, which could increase the risk of infection in wounds, blood, lung, urinary tract, and others. Second, the BICU environment could raise the risk of pathogen transmission. The equipment surfaces, the air, the room walls, and the medical waste in BICU may colonize higher levels of pathogens than those in BCW (Palmieri, 2019; Montazeri et al., 2020). Last, healthcare procedures are perceived as one important route of transmission (AL et al., 2018). Burn patients in BICU may receive multiple invasive procedures, such as central vein/urinary tract catheter, tracheotomy, fiberoptic bronchoscopy, mechanical ventilation, and so on (Tejiram et al., 2019). Furthermore, the intensive care also includes a high frequency of hand contact, and hand hygiene is evident to effectively cut down pathogen spread.

In comparing the current results with our preliminary study (Yali et al., 2014), Gram-negative bacteria is still the main pathogen in BICU and BCW, which is also similar to studies in Southeast China (Li et al., 2018), Iran (Emami et al., 2020), Morocco (Frikh et al., 2018), and Lebanon (Bourgi et al., 2020). However, the ranks of the main pathogens have changed. In BICU, *S. aureus* ranks second in this study, compared with third place in the previous study. In BCW, *A. baumannii* ranked third in this study and sixth in the previous study. In both ICU and BCW, the percentage of *E. coli* decreased, but the percentage of *E. cloacae* increased in this study compared with previous results. In BCW, *C. albicans* is in fifth place in the previous study, but there were no fungi in the top 10 strains. *C. albicans* and *C. tropicalis* were the main fungi in BICU during the 9-year study although *C. albicans* and *Smooth Candida mycoderma* were the main fungi in the preliminary study. Furthermore, the antimicrobial resistance of main pathogens also showed different shifts. The underlying reasons are complicated. The constantly updated principle of antibiotics, strict control of nosocomial infection and new wound treatment methods might lead to the dynamic changes of pathogens. In light of the long period and large sample size, we hold the view that this study could better represent the real infection facts in our burn center.

The sample sources and pathogen distribution by sample type were also different between BICU and BCW. More than 90% of samples were wound secretions and tissues in BCW compared with nearly 50% in BICU. Accordingly, the percentages of blood, sputum, and urine were significantly higher in BICU than in BCW. This could be explained by organ dysfunction being relatively common in severe burn patients in BICU, and invasive inspection and treatment were often performed to support and monitor organ function (Kallinen et al., 2012), which increased the risk of infection of the bloodstream and urinary and respiratory tracts. Because microorganism composition may be different by specimen type (Frikh et al., 2018), we further analyzed the pathogen distribution in different clinical sample types. Although Gram-negative bacteria were predominant in wounds from patients in BICU and BCW, *S. aureus* were the most common bacteria, which is consistent with previous studies (El Hamzaoui et al., 2020; Hubab et al., 2020) and data on the China Antimicrobial Surveillance Network (CHINET) (Hu et al., 2018). This could be partly explained by staphylococcus being the main normal flora in skin, but the number of common Gram-positive bacteria types are lower than those of common Gram-negative bacteria types (Ladhani et al., 2020). Overall, *S. aureus*, *A. baumannii*, and *P. aeruginosa* were the three main bacteria in wounds, blood, sputum, and catheters in patients from BICU and BCW. In the same sample type, the percentage of *A. baumannii* and *P. aeruginosa* were higher in patients from BICU than in patients from BCW, but the percentage of *S. aureus* was higher in patients from BCW than in patients from BICU. Our previous results support the concept that enterogenic infection is prone to occur in severe burn patients in BICU (Li et al., 2017). Furthermore, a recent meta-analysis recognized prior exposure to carbapenems and extended-spectrum cephalosporins, urinary/arterial/venous catheter use, mechanical ventilation, and transfusion as the major modifiable risk factors for Gram-negative infection in burn patients (Vickers et al., 2019). The above risk factors mainly exist in burn patients in BICU.

The drug resistance rate of almost every pathogen was higher in BICU than in BCW. Furthermore, the percentage of MDR bacteria in BICU were higher than those in BCW. However, the gap between BICU and BCW has gradually closed from 2011 to 2019, implying an increasing frequency of MDR in BCW. From 2011 to 2019, the prevalence of MDR *A. baumannii* significantly increased in BICU (80.2% in 2011 vs. 92.7% in 2019) and BCW (59.7% in 2011 vs. 89.3% in 2019). The rate of MDR *K. pneumoniae* also increased in BICU (67.7% in 2015 vs. 86.8% in 2019) and BCW (40.0% in 2015 vs. 80.7% in 2019). The MRSA prevalence of 89.5% to 96.3% in BICU and 62.8% to 82.9% in BCW indicates that the occurrence rate of MRSA stayed at a high level in the burn population. The changing pattern is consistent with the global threaten of MDR organisms. The long-term and combined use of strong antibiotics, increased invasive procedures, and long periods of hospitalization increase the risk of MDR organisms in burn patients (Robben et al., 2020). The high incidence of fungi, which are usually opportunistic pathogens and secondary to long-term use of antibiotics (Zhou et al., 2019), partly support this explanation. Furthermore, the BICU environment might also burden the spread of MDR pathogens. We previously detected MDR *A. baumannii* from our BICU environment, and it possessed similar genotypes to *A. baumannii* from patients in BICU (Gong et al., 2016). The outbreak of MDR bacteria occurs at a rate of 1.9%–66.7% in burn patients worldwide. Studies show that MRSA and *A. baumannii* are the most frequent microbial agents of outbreak and significantly increased morbidity and mortality (Girerd-Genessay et al., 2016). Our BICU also had a small-scale outbreak of *A. baumannii* in 2016 (Gong et al., 2016) and *K. pneumoniae* in 2017 (Gong et al., 2019). However, the rate of MDR *P. aeruginosa* sharply decreased in BICU (85.7% in 2011 to 24.5% in 2019) and BCW (23.5% in 2011 vs. 6.9% in 2019). The reduced prevalence of MDR *P. aeruginosa* is consistent with the nationwide data on CHINET, which mainly was attributed to the strict control of the use of antibiotics, especially high-level, broad-spectrum antibiotics, and the scientific application of antibiotics based on PK/PD parameter. Furthermore, we also decreased the humidity of the ward environment, and the humid environment might increase the spread and growth of *P. aeruginosa.* However, all the management strategies were not enough because the prevalence of other MDR bacteria were still high. In the absence of more effective methods, hand hygiene, use of personal protective equipment, contact isolation, negative-pressure patient rooms, frequent room cleaning, and daily evaluation of invasive lines and devices are necessary to reduce the risk of hospital infection (Tejiram and Shupp, 2020).

In line with several other studies (Vickers et al., 2019; Bourgi et al., 2020; Emami et al., 2020), *A. baumannii*, *P. aeruginosa*, and *K. pneumoniae* are the predominant Gram-negative bacteria in BICU and BCW. Overall, the resistant rate of *A. baumannii* and *K. pneumoniae* gradually increased in BICU and BCW although the resistance rate of *P. aeruginosa* decreased in BICU and stayed low in BCW in accordance with all of China (Hu et al., 2018). However, the curves of different antibiotic resistance rates are tending to unanimity, meaning emerging MDR bacteria. Fortunately, all the Gram-negative bacteria were sensitive to tigecycline and polymyxin B. Prevalence of carbapenem-resistant Gram-negative bacteria has become a major global public health problem because of high mortality and poor effective antibiotics. In our center, the major carbapenem-resistant Gram-negative bacteria were *A. baumannii*, *P. aeruginosa*, and CREs, which include *K. pneumoniae*, *E. coli*, and *E. cloacae*. The carbapenem resistance rate of *A. baumannii* was constantly at a a high level in BICU (>90%) and elevated from about 60% in 2011–2013 to 81.8% in 2019. The carbapenem resistance rate of *P. aeruginosa* in BICU significantly decreased during 2011 to 2019 and was equal to that in BCW in 2019 (about 20%). Furthermore, the carbapenem resistance rate of *K. pneumoniae* significantly increased in both BICU and BCW and was about 40% in 2017–2019. The kinetics of carbapenem-resistant Gram-negative bacteria were similar to data on CHINET (Hu et al., 2018; Hu et al., 2019). However, the carbopenem resistance of *A. baumannii* and *K. pneumoniae* is more severe in our center than in all of China (Hu et al., 2019) (73.6% in *A. baumannii*, 27.5% in *P. aeruginosa*, 25.3% in *K. pneumoniae*). In fact, the destroyed skin barrier and continuous antibiotic treatments not only make burn units the breeding ground for all these MDR organisms, but they also make burn infection more severe and common than others (Shokrollahi and Singleton, 2017). The levofloxacin resistance by *A. baumannii* began to fall in 2015, which was similar to *P. aeruginosa* in BICU. Strict control of the clinical use of levofloxacin mainly contributed to this phenomenon because resistance to levofloxacin was very common and severe before 2015. After 2015, tigecycline, polymyxin B/colistin, beta-lactamase inhibitor, and carbapenem were recommended for the treatment of *A. baumannii* and other Gram-negative bacteria. Furthermore, the change of key resistance genes, such as *gyrA* and *parC*, might also lead to the fall of levofloxacin resistance. However, more research is needed to confirm the gene changes in the future.

The molecular mechanisms of antibiotic resistance are different in different bacteria. The mechanism of antibiotic resistance mainly includes the production of inactive enzymes (β-lactamase and aminoglycoside modifying enzymes), the modification of the target site, the decrease of drug penetrance, and the overexpression of efflux pumps. We previously found that the β-lactamase genes, including *OXA-23*, *AmpC*, *IS-AmpC*, *PER*, *VIM*, and *SIM*, were the five most prevalent resistant genes in MDR *A. baumannii* (Gong et al., 2016; Huang et al., 2016). Furthermore, *ST368* was the dominant genotype (Gong et al., 2016), and *clonal complex 92* (*CC92*) was the primary complex (Huang et al., 2016) in *A. baumannii* subtypes. However, the mutant inactivation of the *oprD* porin gene and overexpression of the *ampC* β-lactamase gene mainly contributed to the carbapenem resistance of *P. aeruginosa* (Yin et al., 2018). The ST genotype was diverse in *P. aeruginosa*. More than 90% of CRKP produces the extensive β-lactamase (*blaCTX-M-10*, *blaSHV*, *blaTEM*, *blaCTX-M-14*), the *β-bla_ACT_*lactamase, and the *bla_KPC_*carbapenemases (Gong et al., 2019). The detection of antibiotic-resistant mechanisms could provide clues for the choice of different antibiotics. In our center, we would further genotype the pathogens from critical patients who need long-term combined antibiotic treatment.

Consistent with previous results (Hu et al., 2018; El Hamzaoui et al., 2020), *S. aureu*s was the most common pathogen in burn wounds, and the percentage of *S. aureus* was higher in BCW than in BICU. However, the percentage of MRSA was significantly higher in BICU (89.5% to 96.3%) than in BCW (62.8% to 82.9%). Fortunately, almost all MRSAs were sensitive to vancomycin, teicoplanin, and linezolid. The production of penicillin-binding proteins, regulated by the *mec A* and *mec C* genes, is mainly responsible for the resistance of *S. aureus* to β-lactam antibiotics. Staphylococcal chromosomal cassette mec *(SCCmec*) typing showed that a majority of MRSA isolates in our center belonged to SCCmec type III, which represents nosocomial infection. Virulence profile analysis shows that most MRSA isolates carried the virulence factor pattern of c*na-clfA-clfB-eno-fib-icaA-icaD-sea-psmα-lukED-hlg-hlgv-hla-hld* (Jiang et al., 2017). The resistant mechanism strengthened the importance of nosocomial infection control. Moreover, wound infection of *S. aureus* could rapidly lead to skin graft dissolution and biofilm formation. Therefore, enough debridement during operation, timely application of effective antibiotics during the perioperative period, and early and regular dressing changes also should be performed to better avoid MRSA infection.

The treatment of MDR bacteria, especially carbapenem-resistant *A. baumannii*, *P. aeruginosa*, and *K. pneumoniae*, have always been a troublesome challenge. As a result of a lack of sensitive antibiotic drugs, combinations of different types of chemical antibiotics became the current strategy (Robben et al., 2020). However, the combined strategy could further increase the cross-resistance of bacteria, and more and more “super bacteria” have emerged. In 2017, the World Health Organization warned of the global threat of MDR Gram-negative pathogens. In recent years, phage therapy has been shown as a promising alternative to conventional antibiotic therapy (Kortright et al., 2019). We previously found that several new lytic phages show effective antimicrobial potential against *A. baumannii* (Yang et al., 2019), *P. aeruginosa* (Li et al., 2017), and *K. pneumoniae* (Shi et al., 2020). However, the clinical application of a 12-phage cocktail on *P. aeruginosa* infected burn wounds showed poor results compared with 1% sulfadiazine silver (Jault et al., 2019). Therefore, more translational research is still needed to optimize the utilization of phage therapy.

Invasive fungal infections are one of the most severe complications in burn patients and associated with poor outcomes (Maurel et al., 2020). Burn wounds are the main sources of fungi in our center, similar to Pakistan (Jabeen et al., 2020). However, the incidence of fungal infection is lower in our center (11.8% in BICU and 8.1% in BCW) than in India (26%) (Sharma et al., 2016) and similar to that in Morocco (10%) (Rafik et al., 2016). *C. albicans* were the most common yeasts in India and Morocco, and *C. tropicalis* was the most common in Pakistan. However, *C. albicans* is the most common in our center. Although the guidelines of fungal infection diagnosis and treatment has been implemented since 2013 (Luo et al., 2014), the diagnosis and treatment of invasive fungal infections are still nonspecific and inadequate. Therefore, it is important to recognize burn patients with high risk factors of fungal infection. Several studies show that large burn area and depth, prolonged broad-spectrum antibiotic therapy, and increased postburn days were risk factors of fungal infections in burn patients (Rafik et al., 2016; Jabeen et al., 2020). Bacterial coinfection and presence of allografts could further increase the mortality of patients with fungal infections (Maurel et al., 2020). In this study, we also found 45.2% and 58.6% of patients with fungal infection complicated with bacterial infection in BCW and BICU, respectively. Our previous study showed that 54.63% of major burn patients with candidemia had bacteremia (Zhou et al., 2019). Therefore, our results, in part, support that bacterial coinfection could increase the risk of fungal infection. Further clinical investigations with large sample sizes in multiple centers are still required to confirm these findings. Our results also found that amphotericin B was the most effective agent for fungi, followed by voriconazole and fluconazole. However, the susceptibility rate of *non-albicans candida* to voriconazole and fluconazole significantly decreased. Unfortunately, we have not routinely detected a sensitivity to echinocandins. Amphotericin B and voriconazole were the antifungal drugs used most commonly in our center.

This study also has some limitations. First, this is a retrospective study in one burn center, and the results should be carefully interpreted. However, pathogen characteristics may differ by time, center, and country. Therefore, the results in this study could only reflect the pathogen feature in our burn center. Second, this study only included the first positive results, which follows standard international practices. In fact, pathogens in one patient may change over time and after antibiotic exposure (D’Abbondanza and Shahrokhi, 2020). The results of this study may only provide evidence of initial empiric antibiotic therapy. The timely and repeated detection of microbiology in different samples are always necessary and most important. Third, this study mainly focuses on the difference of infection pattern between BICU and BCW. The occurrence of burn infection is a multifactorial event. The influence of other factors, such as injury timeline, burn area, age, and others on infection profile were out of scope of this study and will be investigated in future.

In conclusion, this study further confirms that infection profile shows different patterns between burn patients in BICU and BCW. Pathogen distribution also differed by sample sources. Lower percentages of Gram-positive bacteria and higher percentages of Gram-negative bacteria and fungi were found in BICU than in BCW. *A. baumannii*, *S. aureus*, and *P. aeruginosa* were the most common pathogens although the ranks were different in BICU and BCW. Furthermore, the drug-resistance rates of almost every pathogen were higher in BICU than in BCW, and the MDR bacteria, especially the CREs, became a clear and serious threat in recent years. The occurrence rate of MRSA stayed at a high level in BICU. Regarding the different features of microbiological epidemiology between BICU and BCW, different target strategies of infection control and prevention should be formulated and implemented for different burn populations.

## Data Availability Statement

The original contributions presented in the study are included in the article/**Supplementary Material**. Further inquiries can be directed to the corresponding author.

## Ethics Statement

The studies involving human participants were reviewed and approved by the ethical committee of the Southwest Hospital (NO. KY201991). Written informed consent from the participants’ legal guardian/next of kin was not required to participate in this study in accordance with the national legislation and the institutional requirements.

## Author Contributions

YG: acquisition of data, drafting the article, the conception and design of the study, final approval of the version to be submitted. YAP: acquisition of data, final approval of the version to be submitted. XL: acquisition of data, final approval of the version to be submitted. CZ: acquisition of data, final approval of the version to be submitted. YS: acquisition of data, final approval of the version to be submitted. YZ: analysis and interpretation of data, final approval of the version to be submitted. JD: analysis and interpretation of data, final approval of the version to be submitted. YZP: analysis and interpretation of data, the conception and design of the study, final approval of the version to be submitted. GL: analysis and interpretation of data, final approval of the version to be submitted. HL: the conception and design of the study, revising the article critically for important intellectual content, final approval of the version to be submitted. All authors contributed to the article and approved the submitted version.

## Funding

This study was supported by grants from National Natural Science Foundation of China (82002036) and Military Medical Science & Technology Youth Program (20QNPY011). The funders had no role in study design, data collection and interpretation, or the decision to submit the manuscript for publication.

## Conflict of Interest

The authors declare that the research was conducted in the absence of any commercial or financial relationships that could be construed as a potential conflict of interest.
