# Supplementary material for: Different Infection Profiles and Antimicrobial Resistance Patterns Between Burn ICU and Common Wards
Source: Front Cell Infect Microbiol. 2021 Jun 30;11:681731. doi: 10.3389/fcimb.2021.681731 (PMC8278283; doi:10.3389/fcimb.2021.681731)
Supplement: Supplementary file 1 [file DataSheet_1.doc]

**Supplementary tables**

|  | **2011** | | **2012** | | **2013** | | **2014** | | **2015** | | **2016** | | **2017** | | **2018** | | **2019** | |
| --- | --- | --- | --- | --- | --- | --- | --- | --- | --- | --- | --- | --- | --- | --- | --- | --- | --- | --- |
| **Pathogens** | **Stain**  **(n)** | **Percentage（%）** | **Stain**  **(n)** | **Percentage（%）** | **Stain**  **(n)** | **Percentage（%）** | **Stain**  **(n)** | **Percentage（%）** | **Stain**  **(n)** | **Percentage（%）** | **Stain**  **(n)** | **Percentage（%）** | **Stain**  **(n)** | **Percentage（%）** | **Stain**  **(n)** | **Percentage（%）** | **Stain**  **(n)** | **Percentage（%）** |
| ***A. baumannii*** | 62 | 21.8 | 71 | 16.7 | 94 | 17.1 | 110 | 21.7 | 78 | 16.5 | 68 | 21.1 | 70 | 22.8 | 65 | 18.7 | 42 | 17.6 |
| ***S. aureus*** | 45 | 15.8 | 62 | 14.6 | 83 | 15.1 | 95 | 18.7 | 87 | 18.4 | 63 | 19.5 | 56 | 18.2 | 65 | 18.7 | 50 | 20.9 |
| ***P. aeruginosa*** | 45 | 15.8 | 60 | 14.6 | 68 | 12.3 | 75 | 14.8 | 84 | 17.8 | 43 | 13.3 | 38 | 12.4 | 44 | 12.7 | 35 | 14.6 |
| ***K. pneumoniae*** | 22 | 7.7 | 32 | 7.5 | 40 | 7.3 | 23 | 4.5 | 19 | 4.0 | 19 | 5.9 | 35 | 11.4 | 26 | 7.5 | 12 | 5.0 |
| ***E. cloacae*** | 6 | 2.1 | 15 | 3.5 | 31 | 5.6 | 22 | 4.3 | 18 | 3.8 | 11 | 3.4 | 4 | 1.3 | 9 | 2.6 | 3 | 1.3 |
| ***C. albicans*** | 8 | 2.8 | 19 | 4.5 | 21 | 3.8 | 11 | 2.2 | 11 | 2.3 | 9 | 2.8 | 8 | 2.6 | 7 | 2.0 | 7 | 2.9 |
| ***E. coli*** | 11 | 3.9 | 22 | 5.2 | 21 | 3.8 | 17 | 3.3 | 19 | 4.0 | 15 | 4.6 | 12 | 3.9 | 13 | 3.7 | 8 | 3.3 |
| ***E. faecium*** | 6 | 2.1 | 6 | 1.4 | 20 | 3.6 | 8 | 1.6 | 15 | 3.2 | 8 | 2.5 | 5 | 1.6 | 10 | 2.9 | 3 | 1.3 |
| ***S. haemolyticus*** | 1 | 0.4 | 6 | 1.4 | 20 | 3.6 | 16 | 3.1 | 18 | 3.8 | 5 | 1.5 | 6 | 2.0 | 4 | 1.2 | 3 | 1.3 |
| ***C. tropicalis*** | 5 | 1.8 | 5 | 1.2 | 18 | 3.3 | 13 | 2.6 | 14 | 3.0 | 4 | 1.2 | 8 | 3.6 | 10 | 2.9 | 10 | 4.2 |
| ***S. maltophilia*** | 8 | 2.8 | 8 | 1.9 | 12 | 2.2 | 13 | 2.6 | 18 | 3.8 | 10 | 3.1 | 12 | 3.9 | 12 | 3.5 | 8 | 3.3 |
| Total | 219 | 77.0 | 306 | 72.5 | 428 | 77.7 | 403 | 79.4 | 381 | 80.6 | 255 | 78.9 | 254 | 83.7 | 265 | 76.4 | 181 | 75.7 |

**Table S1 Annual distribution of top-11 pathogens form burn ICU**

**Table S2 Annual distribution of top-11 pathogens form burn common wards**

|  | **2011** | | | **2012** | | **2013** | | **2014** | | **2015** | | **2016** | | **2017** | | **2018** | | **2019** | |
| --- | --- | --- | --- | --- | --- | --- | --- | --- | --- | --- | --- | --- | --- | --- | --- | --- | --- | --- | --- |
| **Pathogens** | **Stain**  **(n)** | | **Percentage（%）** | **Stain**  **(n)** | **Percentage（%）** | **Stain**  **(n)** | **Percentage（%）** | **Stain**  **(n)** | **Percentage（%）** | **Stain**  **(n)** | **Pathogens** | **Stain**  **(n)** | **Percentage（%）** | **Stain**  **(n)** | **Percentage（%）** | **Stain**  **(n)** | **Percentage（%）** | **Stain**  **(n)** | **Percentage（%）** |
| ***S. aureus*** | | 40 | 23.5 | 79 | 24.6 | 167 | 23.1 | 229 | 24.7 | 195 | 27.0 | 147 | 35.6 | 72 | 28.8 | 94 | 28.8 | 76 | 24.0 |
| ***P. aeruginosa*** | | 24 | 14.1 | 45 | 14.0 | 78 | 10.8 | 124 | 13.4 | 121 | 16.8 | 61 | 14.8 | 46 | 15.4 | 68 | 20.9 | 63 | 19.7 |
| ***A. baumannii*** | | 8 | 4.7 | 23 | 7.2 | 52 | 7.2 | 67 | 7.2 | 36 | 5.0 | 35 | 8.5 | 22 | 7.4 | 23 | 7.1 | 22 | 6.9 |
| ***K. pneumoniae*** | | 12 | 7.1 | 21 | 6.5 | 46 | 6.4 | 37 | 4.0 | 25 | 3.5 | 16 | 3.9 | 15 | 5.0 | 9 | 2.8 | 5 | 1.6 |
| ***E. coli*** | | 12 | 7.1 | 22 | 6.9 | 57 | 7.9 | 54 | 5.8 | 45 | 6.2 | 26 | 6.3 | 22 | 7.4 | 10 | 3.1 | 17 | 5.3 |
| ***E. cloacae*** | | 4 | 2.4 | 12 | 3.7 | 51 | 7.1 | 63 | 6.8 | 34 | 4.7 | 11 | 2.7 | 13 | 4.4 | 10 | 3.1 | 16 | 5.0 |
| ***S. haemolyticus*** | | 4 | 2.4 | 12 | 3.7 | 29 | 4.0 | 37 | 4.0 | 31 | 4.3 | 13 | 3.1 | 10 | 3.4 | 5 | 1.5 | 7 | 2.2 |
| ***P. mirabilis*** | | 4 | 2.4 | 8 | 2.5 | 16 | 2.2 | 36 | 3.9 | 17 | 2.4 | 12 | 2.9 | 13 | 4.4 | 5 | 1.5 | 8 | 2.5 |
| ***E. faecium*** | | 2 | 1.2 | 9 | 2.8 | 20 | 2.8 | 29 | 3.1 | 29 | 4.0 | 11 | 2.7 | 7 | 2.3 | 10 | 3.1 | 8 | 2.5 |
| ***S. hominis*** | | 6 | 3.5 | 2 | 0.6 | 13 | 1.8 | 20 | 2.2 | 8 | 1.1 | 8 | 1.9 | 4 | 1.3 | 7 | 2.1 | 6 | 1.9 |
| ***S.maltophilia*** | | 8 | 4.7 | 4 | 1.1 | 10 | 1.4 | 11 | 1.2 | 13 | 1.8 | 3 | 0.7 | 3 | 1.0 | 5 | 1.5 | 3 | 0.9 |
| **Total** | 124 | | 73.1 | 237 | 73.6 | 539 | 74.7 | 707 | 76.3 | 554 | 76.8 | 343 | 83.1 | 227 | 80.8 | 246 | 75.5 | 231 | 72.5 |

**Table S3 Comparison of the resistance rate of *A. baumannii* from burn ICU and common wards during 2011 to 2019**

BICU: Burn intensive care units; BCW: burn common wards; ND: Not done.

|  | 2011 | | 2012 | | 2013 | | 2014 | | 2015 | | 2016 | | 2017 | | 2018 | | 2019 | |
| --- | --- | --- | --- | --- | --- | --- | --- | --- | --- | --- | --- | --- | --- | --- | --- | --- | --- | --- |
|  | BICU  (%) | BCW  (%) | BICU  (%) | BCW  (%) | BICU  (%) | BCW  (%) | BICU  (%) | BCW  (%) | BICU  (%) | BCW  (%) | BICU  (%) | BCW  (%) | BICU  (%) | BCW  (%) | BICU  (%) | BCW  (%) | BICU  (%) | BCW  (%) |
| Cefoperazone/Subactam | 76.9 | 59.2 | 78.9 | 60.9 | 56.4 | 78.3 | 80.0 | 52.2 | 81.8 | 61.6 | 78.6 | 87.5 | 95.7 | 77.3 | 83.1 | 69.6 | 95.2 | 77.3 |
| Piperacillin/tazobactam | 87.5 | 77.4 | 91.5 | 78.3 | 91.5 | 69.2 | 94.5 | 71.6 | 87.2 | 61.6 | 89.1 | 77.3 | 98.6 | 77.3 | 86.2 | 77.3 | 97.6 | 80.0 |
| Imipenem | 83.8 | 59.1 | 85.9 | 60.9 | 86.0 | 61.5 | 87.3 | 55.2 | 85.9 | 58.3 | 91.3 | 77.3 | 98.6 | 77.3 | 85.9 | 77.3 | 97.6 | 81.8 |
| Cefepime | 90.5 | 80.4 | 94.4 | 82.6 | 91.5 | 69.2 | 95.5 | 71.6 | 89.7 | 69.4 | 91.3 | 90.9 | 98.6 | 77.3 | 86.2 | 77.3 | 97.6 | 81.8 |
| Gentamicin | 89.4 | 81.2 | 94.4 | 82.6 | 91.5 | 71.2 | 93.6 | 74.6 | 88.5 | 90.9 | 89.1 | 92.3 | 97.1 | 72.7 | 81.5 | 77.3 | 57.1 | 72.7 |
| Levofloxacin | 86.7 | 77.0 | 88.7 | 78.3 | 91.5 | 69.2 | 93.6 | 67.2 | 87.2 | 91.5 | 60.9 | 59.1 | 51.4 | 54.5 | 56.9 | 36.4 | 21.4 | 31.8 |
| Tigecycline | ND | ND | ND | ND | ND | ND | ND | ND | 0 | 0 | 0 | 0 | 0 | 0 | 0 | 0 | 0 | 0 |
| Polymyxin B | 0 | 0 | 0 | 0 | 0 | 0 | 0 | 0 | 0 | 0 | 0 | 0 | 0 | 0 | 0 | 0 | 0 | 0 |

**Table S4 Comparison of the resistance rate of *P. aeruginosa* from burn ICU and common wards during 2011 to 2019**

|  | 2011 | | 2012 | | 2013 | | 2014 | | 2015 | | 2016 | | 2017 | | 2018 | | 2019 | |
| --- | --- | --- | --- | --- | --- | --- | --- | --- | --- | --- | --- | --- | --- | --- | --- | --- | --- | --- |
|  | BICU  (%) | BCW  (%) | BICU  (%) | BCW  (%) | BICU  (%) | BCW  (%) | BICU  (%) | BCW  (%) | BICU  (%) | BCW  (%) | BICU  (%) | BCW  (%) | BICU  (%) | BCW  (%) | BICU  (%) | BCW  (%) | BICU  (%) | BCW  (%) |
| Cefoperazone/Subactam | 53.1 | 20.0 | 56.9 | 22.2 | 42.6 | 12.8 | 70.3 | 21.3 | 36.9 | 21.5 | 25.9 | 18.4 | 15.4 | 15.9 | 7.1 | 4.4 | 22.2 | 23.8 |
| Piperacillin/tazobactam | 50.2 | 22.5 | 56.7 | 24.4 | 50.0 | 15.4 | 72.0 | 18.5 | 29.8 | 20.7 | 28.6 | 20.0 | 7.7 | 17.4 | 9.3 | 4.4 | 24.4 | 14.5 |
| Cefepime | 53.1 | 32.1 | 61.7 | 33.3 | 58.8 | 17.9 | 69.3 | 18.5 | 53.6 | 32.2 | 20.6 | 28.8 | 7.7 | 19.6 | 11.4 | 4.5 | 33.3 | 23.3 |
| Imipenem | 62.2 | 19.5 | 66.7 | 20.0 | 36.8 | 9.0 | 58.7 | 16.5 | 42.9 | 25.8 | 33.3 | 31.4 | 41.7 | 11.4 | 21.2 | 7.3 | 20.0 | 20.8 |
| Gentamicin | 84.5 | 23.0 | 85.0 | 22.2 | 73.5 | 30.8 | 69.3 | 26.0 | 52.4 | 31.4 | 14.7 | 13.3 | 16.7 | 24.4 | 4.7 | 6.0 | 22.2 | 16.1 |
| Levofloxacin | 66.1 | 19.6 | 68.3 | 20.0 | 61.8 | 20.5 | 52.0 | 21.0 | 34.5 | 26.4 | 14.3 | 33.3 | 23.1 | 11.4 | 2.3 | 1.5 | 20.0 | 16.4 |
| Polymyxin B | 0 | 0 | 0 | 0 | 0 | 0 | 0 | 0 | 0 | 0 | 0 | 0 | 0 | 0 | 0 | 0 | 0 | 0 |

BICU: Burn intensive care units; BCW: burn common wards.

|  | 2011 | | 2012 | | | 2013 | | | | 2014 | | | 2015 | | | 2016 | | | 2017 | | | 2018 | | | 2019 | |
| --- | --- | --- | --- | --- | --- | --- | --- | --- | --- | --- | --- | --- | --- | --- | --- | --- | --- | --- | --- | --- | --- | --- | --- | --- | --- | --- |
|  | BICU  (%) | BCW  (%) | | BICU  (%) | BCW  (%) | | BICU  (%) | BCW  (%) | BICU  (%) | | BCW  (%) | BICU  (%) | | BCW  (%) | BICU  (%) | | BCW  (%) | BICU  (%) | | BCW  (%) | BICU  (%) | | BCW  (%) | BICU  (%) | | BCW  (%) |
| Cefoperazone/Subactam | 0 | 0 | | 0 | 0 | | 7.5 | 4.3 | 39.1 | | 5.4 | 21.1 | | 8.0 | 44.4 | | 15.4 | 68.8 | | 53.3 | 12.5 | | 50.0 | 41.7 | | 40.0 |
| Piperacillin/tazobactam | 30.0 | 0 | | 34.4 | 4.8 | | 20.0 | 17.4 | 43.5 | | 24.3 | 10.5 | | 4.0 | 37.5 | | 15.4 | 68.6 | | 40.0 | 19.2 | | 62.5 | 55.8 | | 40.0 |
| Ceftazidime | 54.5 | 33.3 | | 65.6 | 38.1 | | 42.5 | 19.6 | 60.9 | | 27.0 | 42.1 | | 20.0 | 42.9 | | 25.0 | 100 | | 42.9 | 33.3 | | 50.0 | 50.0 | | 100 |
| Cefepime | 59.1 | 66.7 | | 75.0 | 42.9 | | 62.5 | 43.5 | 87.0 | | 37.8 | 52.6 | | 28.0 | 47.1 | | 23.1 | 68.6 | | 53.3 | 30.8 | | 50.0 | 83.3 | | 60.0 |
| Aztreonam | 63.6 | 66.7 | | 68.8 | 52.4 | | 57.5 | 39.1 | 95.7 | | 40.5 | 52.6 | | 28.0 | 52.9 | | 30.8 | 67.6 | | 57.1 | 38.5 | | 50.0 | 41.7 | | 80.0 |
| Imipenem | 4.5 | 0 | | 3.1 | 0 | | 0 | 0 | 8.7 | | 8.1 | 10.5 | | 0 | 35.3 | | 15.4 | 62.9 | | 40.0 | 16.0 | | 50.0 | 40.0 | | 40.0 |
| Amikacin | 57.1 | 33.3 | | 59.4 | 28.6 | | 22.5 | 13.0 | 34.8 | | 13.5 | 10.5 | | 12.0 | 37.5 | | 23.1 | 51.4 | | 33.3 | 11.5 | | 25.0 | 50.0 | | 20.0 |
| Gentamicin | 81.8 | 83.3 | | 81.2 | 38.1 | | 47.5 | 43.5 | 73.9 | | 37.8 | 47.4 | | 16.0 | 64.7 | | 46.2 | 77.1 | | 73.3 | 42.3 | | 50.0 | 75.0 | | 40.0 |
| Levofloxacin | 10 | 0 | | 34.4 | 19 | | 32.5 | 21.7 | 39.1 | | 18.9 | 31.6 | | 12.0 | 52.9 | | 30.8 | 68.6 | | 53.3 | 34.6 | | 25.0 | 41.7 | | 40.0 |
| Tigecycline | ND | ND | | ND | ND | | ND | ND | ND | | ND | 0 | | 0 | 0 | | 0 | 0 | | 0 | 0 | | 0 | 0 | | 0 |

**Table S5 Comparison of the resistance rate of *K. pneumoniae* from burn ICU and common wards during 2011 to 2019**

BICU: Burn intensive care units; BCW: burn common wards; ND: Not done.

**Table S6 Comparison of the resistance rate of *S. aureus* from burn ICU and common wards during 2011 to 2019**

|  | 2011 | | 2012 | | 2013 | | 2014 | | 2015 | | 2016 | | 2017 | | 2018 | | 2019 | |
| --- | --- | --- | --- | --- | --- | --- | --- | --- | --- | --- | --- | --- | --- | --- | --- | --- | --- | --- |
|  | BICU  (%) | BCW  (%) | BICU  (%) | BCW  (%) | BICU  (%) | BCW  (%) | BICU  (%) | BCW  (%) | BICU  (%) | BCW  (%) | BICU  (%) | BCW  (%) | BICU  (%) | BCW  (%) | BICU  (%) | BCW  (%) | BICU  (%) | BCW  (%) |
| Penicillin | 100 | 100 | 100 | 98.7 | 100 | 99.4 | 100 | 98.3 | 100 | 95.9 | 100 | 95.3 | 100 | 93.1 | 100 | 97.8 | 100 | 97.2 |
| Oxacillin | 100 | 94.7 | 100 | 93.7 | 100 | 88 | 95.8 | 78.2 | 93.1 | 87.7 | 89.4 | 67.1 | 96.4 | 69 | 94.9 | 68.8 | 95.9 | 81.4 |
| Gentamycin | 100 | 68.4 | 96.8 | 69.6 | 97.6 | 79.6 | 98.9 | 62.4 | 86.2 | 56.4 | 87.2 | 60.5 | 94.6 | 63.9 | 96.9 | 66.0 | 98.0 | 78.9 |
| Rifampicin | 100 | 50.0 | 96.8 | 59.5 | 95.2 | 66.5 | 91.6 | 55.5 | 79.3 | 45.6 | 85.1 | 50.6 | 94.6 | 55.6 | 93.8 | 56.4 | 96.0 | 67.1 |
| Levofloxacin | 100 | 50.0 | 98.4 | 62.0 | 95.2 | 71.3 | 93.7 | 56.8 | 80.5 | 49.7 | 87.2 | 53.5 | 94.6 | 56.9 | 95.4 | 61.7 | 96.0 | 64.5 |
| SMZ-TMP | 4.0 | 5.3 | 1.6 | 7.6 | 6.0 | 9.6 | 3.2 | 9.2 | 3.4 | 5.6 | 4.3 | 7.1 | 0 | 8.3 | 3.1 | 12.8 | 2.0 | 8.0 |
| Clindamycin | 28.0 | 42.1 | 12.9 | 29.1 | 28.9 | 34.7 | 10.5 | 30.1 | 16.1 | 33.3 | 31.6 | 43.3 | 20.0 | 36.7 | 50.0 | 61.1 | 66.7 | 44.4 |
| Erythromycin | 56.0 | 63.2 | 56.5 | 54.4 | 63.9 | 65.3 | 49.5 | 57.2 | 28.7 | 42.6 | 74.5 | 62.4 | 98.2 | 75 | 96.9 | 85.1 | 100 | 77.3 |
| Linezolid | 0 | 0 | 0 | 0 | 0 | 0 | 0 | 0 | 0 | 0 | 0 | 0 | 0 | 0 | 0 | 0 | 0 | 0 |
| Vancomycin | 0 | 0 | 0 | 0 | 0 | 0 | 0 | 0 | 0 | 0 | 0 | 0 | 0 | 0 | 0 | 0 | 0 | 0 |
| Teicoplanin | 0 | 0 | 0 | 0 | 0 | 0 | 0 | 0 | 0 | 0 | 0 | 0 | 0 | 0 | 0 | 0 | 0 | 0 |
| Tetracycline | 92.0 | 68.4 | 98.4 | 73.4 | 98.8 | 80.1 | 93.7 | 64.5 | 86.2 | 62.1 | 87.2 | 64.7 | 98.2 | 59.7 | 96.9 | 71.3 | 96.0 | 72.4 |

BICU: Burn intensive care units; BCW: burn common wards.

**Table S7 Comparison of the resistance rate of *S. haemolyticus* from burn ICU and common wards during 2011 to 2019**

|  | 2011 | | 2012 | | 2013 | | 2014 | | 2015 | | 2016 | | 2017 | | 2018 | | 2019 | |
| --- | --- | --- | --- | --- | --- | --- | --- | --- | --- | --- | --- | --- | --- | --- | --- | --- | --- | --- |
|  | BICU  (%) | BCW  (%) | BICU  (%) | BCW  (%) | BICU  (%) | BCW  (%) | BICU  (%) | BCW  (%) | BICU  (%) | BCW  (%) | BICU  (%) | BCW  (%) | BICU  (%) | BCW  (%) | BICU  (%) | BCW  (%) | BICU  (%) | BCW  (%) |
| Penicillin | 100 | 100 | 100 | 100 | 100 | 100 | 100 | 93.8 | 100 | 100 | 100 | 100 | 100 | 100 | 100 | 100 | 100 | 100 |
| Oxacillin | 100 | 100 | 100 | 100 | 96.6 | 100 | 100 | 93.8 | 96.8 | 100 | 100 | 100 | 100 | 100 | 100 | 75 | 85.7 | 100 |
| Gentamycin | 100 | 100 | 66.7 | 83.3 | 82.8 | 100 | 86.5 | 93.8 | 71 | 83.3 | 100 | 75 | 50 | 100 | 100 | 75 | 57.1 | 66.7 |
| Rifampicin | 0 | 100 | 16.7 | 16.7 | 31 | 30 | 37.8 | 37.5 | 29 | 33.3 | 62.5 | 25 | 30 | 0 | 0 | 50 | 28.6 | 33.3 |
| Levofloxacin | 100 | 100 | 72.7 | 83.3 | 79.3 | 90 | 89.2 | 93.8 | 90.3 | 100 | 100 | 100 | 90 | 100 | 100 | 75 | 100 | 100 |
| SMZ-TMP | 100 | 0 | 50 | 83.3 | 55.2 | 60 | 62.2 | 62.5 | 64.5 | 61.1 | 42.9 | 75 | 50 | 16.7 | 40 | 50 | 71.4 | 66.7 |
| Clindamycin | 100 | 100 | 41.7 | 83.3 | 58.6 | 80 | 78.4 | 75 | 64.5 | 77.8 | 87.5 | 100 | 80 | 66.7 | 66.7 | 0 | 60 | 0 |
| Erythromycin | 100 | 100 | 91.7 | 83.3 | 100 | 100 | 100 | 93.8 | 90.3 | 88.9 | 100 | 100 | 100 | 100 | 100 | 100 | 100 | 100 |
| Linezolid | 0 | 0 | 0 | 0 | 0 | 0 | 0 | 0 | 0 | 0 | 0 | 0 | 0 | 0 | 0 | 0 | 0 | 0 |
| Vancomycin | 0 | 0 | 0 | 0 | 0 | 0 | 0 | 0 | 0 | 0 | 0 | 0 | 0 | 0 | 0 | 0 | 0 | 0 |
| Teicoplanin | 0 | 0 | 0 | 0 | 0 | 0 | 0 | 0 | 0 | 0 | 0 | 0 | 0 | 0 | 0 | 0 | 0 | 0 |
| Tetracycline | 0 | 0 | 8.3 | 16.7 | 20.7 | 25 | 45.9 | 60 | 32.3 | 50 | 14.3 | 50 | 40 | 50 | 60 | 50 | 42.9 | 66.7 |

BICU: Burn intensive care units; BCW: burn common wards.

**Table S8 Comparison of the resistance rate of *E. faecium* from burn ICU and common wards during 2011 to 2019**

|  | 2011 | | 2012 | | 2013 | | 2014 | | 2015 | | 2016 | | 2017 | | 2018 | | 2019 | |
| --- | --- | --- | --- | --- | --- | --- | --- | --- | --- | --- | --- | --- | --- | --- | --- | --- | --- | --- |
|  | BICU  (%) | BCW  (%) | BICU  (%) | BCW  (%) | BICU  (%) | BCW  (%) | BICU  (%) | BCW  (%) | BICU  (%) | BCW  (%) | BICU  (%) | BCW  (%) | BICU  (%) | BCW  (%) | BICU  (%) | BCW  (%) | BICU  (%) | BCW  (%) |
| Ampicillin | 81.4 | 100 | 83.3 | 92.3 | 50 | 90.9 | 75 | 88.2 | 50 | 70 | 57.6 | 66.7 | 100 | 100 | 50 | 80 | 50 | 66.7 |
| Gentamycin | 63.0 | 75 | 83.3 | 41.7 | 60 | 54.5 | 40 | 87.5 | 60 | 70 | 31.9 | 33.3 | 50 | 25 | 50 | 80 | 50 | 33.3 |
| Rifampicin | 81.4 | 100 | 83.3 | 92.3 | 70 | 90.9 | 58.3 | 82.4 | 80 | 90 | 0 | 0 | ND | ND | ND | ND | ND | ND |
| Ciprofloxacin | 81.4 | 100 | 50 | 92.3 | 50 | 81.8 | 41.7 | 88.2 | 50 | 80 | 57.6 | 66.7 | 100 | 100 | 50 | 80 | 50 | 66.7 |
| Moxifloxacin | ND | ND | ND | ND | ND | ND | ND | ND | ND | ND | ND | 66.7 | 100 | 100 | 50 | 80 | 100 | 66.7 |
| Erythromycin | 81.4 | 100 | 83.3 | 92.3 | 80 | 90.9 | 83.3 | 100 | 90 | 100 | 48.6 | 50 | 100 | 100 | 100 | 100 | 50 | 100 |
| Linezolid | 0 | 0 | 0 | 0 | 0 | 0 | 0 | 0 | 0 | 0 | 0 | 0 | 0 | 0 | 0 | 0 | 0 | 0 |
| Vancomycin | 0 | 0 | 0 | 0 | 0 | 0 | 0 | 0 | 0 | 0 | 0 | 0 | 0 | 0 | 0 | 0 | 0 | 0 |
| Teicoplanin | 0 | 0 | 0 | 0 | 0 | 0 | 0 | 0 | 0 | 0 | 0 | 0 | 0 | 0 | 0 | 0 | 0 | 0 |
| Tetracycline | 43.9 | 50 | 66.7 | 38.5 | 70 | 81.8 | 41.7 | 64.7 | 70 | 70 | 63.9 | 100 | 50 | 100 | 0 | 90 | 0 | 33.3 |

BICU: Burn intensive care units; BCW: burn common wards; ND: Not done.
